# Relative Impact of GLP-1 Agonist Use on Microvascular Versus Macrovascular Complications

**DOI:** 10.7759/cureus.93925

**Published:** 2025-10-06

**Authors:** Efeturi M Okorigba, Kehinde H Jinadu, Oto-obong J Utuk, Akinyemi Akinwumiju, Olasunkanmi A Kolawole, Fatimot Disu, Victor Sosu

**Affiliations:** 1 Internal Medicine, West Virginia University, Morgantown, USA; 2 Internal Medicine, Spire Leeds Hospital, Leeds, GBR; 3 General Medicine, Windsor University School of Medicine, Mississauga, CAN; 4 Psychiatry and Behavioral Sciences, Progressive Psychiatric Services, Las Vegas, USA; 5 Internal Medicine, University of Texas School of Public Health, Houston, USA; 6 General Internal Medicine, Salisbury NHS Foundation Trust, Salisbury , GBR; 7 Nephrology, Lenox Hill Hospital, New York, USA

**Keywords:** albuminuria, diabetes mellitus, glucagon-like peptide-1 receptor agonists, macrovascular complications, microvascular complications

## Abstract

Background: Diabetes and obesity contribute to vascular complications. The effects of glucagon-like peptide-1 receptor agonists (GLP-1 RAs) on microvascular and macrovascular outcomes in the general population remain less well understood.

Objective: To compare the adjusted odds of microvascular and macrovascular complications among adults in the United States (US) using GLP-1 RAs.

Methods: We conducted a survey-weighted logistic regression analysis using National Health and Nutrition Examination Survey (NHANES) data from 2011-2018. Microvascular complications were defined as albuminuria or diabetic retinopathy, while macrovascular complications included myocardial infarction, stroke, or coronary artery disease. Models adjusted for demographic, socioeconomic, and clinical factors.

Results: In the adjusted models, the use of GLP-1 RA was linked to increased odds of microvascular complications (OR 2.29, 95% CI 1.05-4.97, p=0.037). No significant association was observed with macrovascular complications (OR 1.27, 95% CI 0.65-2.51, p=0.478). Established risk factors, including older age, higher BMI, lower income, and smoking, were independently associated with higher odds of vascular complications.

Conclusion: The use of GLP-1 RAs was linked to increased odds of microvascular complications following adjustment for the confounders, even as no significant association was reported with macrovascular complications. This possibly reflects confounding through indication, given that such medications/agents are mainly prescribed to persons with either longer duration or increasingly severe diabetes. These findings indicate the need for longitudinal studies to explain the temporal correlations between the use of GLP-1 RA and complication risk.

## Introduction

Diabetes mellitus and obesity are some of the major causes of morbidity and mortality in the United States, and they are the major contributors to the burden of vascular complications [1]. The Centers for Disease Control and Prevention report that there are over 37 million adults in the United States living with diabetes, and over 42% of adults are considered obese [2]. The two conditions are closely linked to vascular complications affecting quality of life and raising health care expenditures [3, 4]. For instance, individuals with diabetes have 2-4 times higher risk of cardiovascular disease (CVD), even as the global healthcare costs associated with obesity are forecasted to increase to $3 trillion yearly by 2030 [1-5]. These complications are conventionally divided into macrovascular complications, including myocardial infarction, stroke, and coronary artery disease, and microvascular complications, including chronic kidney disease (CKD), albuminuria, and diabetic retinopathy [5]. Although the difference is clinically helpful, both groups have common pathophysiologic mechanisms of hyperglycemia-mediated endothelial dysfunction, oxidative stress, and inflammation [6]. It is noteworthy that effective management of obesity and diabetes mellitus alongside their complications is influenced by the intricate interplay of socioeconomic and clinical factors. For example, persons with lower socioeconomic status and those with specific comorbidities, including congestive heart failure and HIV (human immunodeficiency virus), normally experience significant barriers to care, including the reduced likelihood of filling for novel therapy prescriptions such as glucagon-like peptide-1 receptor agonists (GLP-1 RAs). This might result in suboptimal treatment adherence and eventually contribute to worse clinical care outcomes, disproportionately affecting such vulnerable populations [3-7].

Macrovascular complications are frequently emphasized due to being the leading cause of death in diabetes and obesity, and the cause of most cardiovascular deaths [7]. The results of large cardiovascular outcome trials (CVOTs) have repeatedly shown that patients with diabetes are at a two- to four-fold higher risk of atherosclerotic events than non-diabetic patients [8]. Such hard clinical endpoints, including stroke and myocardial infarction, are also increasingly readily measured in the large-scale studies. Microvascular complications, on the other hand, are more insidious, usually developing earlier in the course of the disease and playing a significant role in morbidity [9]. CKD, albuminuria, and retinopathy contribute to reduced quality of life, functional impairment, and, in the case of advanced kidney disease, increased cardiovascular risk and premature death [10]. Despite this burden, microvascular endpoints have traditionally been less emphasized in clinical trials than macrovascular outcomes [11].

The introduction of GLP-1 RAs has dramatically changed the treatment of type 2 diabetes and, more recently, obesity [12]. Designed initially as antihyperglycemic agents, GLP-1 RAs, including liraglutide, semaglutide, and dulaglutide, have a variety of effects beyond glucose lowering, such as appetite suppression, weight loss, small blood pressure reductions, and lipid metabolism improvements [13]. Notably, multiple large CVOTs have shown that GLP-1 RAs lower the risk of major adverse cardiovascular events (MACE), especially non-fatal stroke and cardiovascular death, in patients with diabetes and elevated baseline cardiovascular risks [14].

There is some evidence that GLP-1 RAs can decrease albuminuria and retard CKD progression, possibly through anti-inflammatory and natriuretic effects [15]. However, retinopathy outcome data have been inconsistent, with some trials indicating potential early exacerbation of retinopathy with rapid glycemic improvement [16, 17]. With the increasing use of GLP-1 RAs in populations outside of diabetes, especially in managing obesity, understanding these differential effects is increasingly clinically and publicly relevant [18].

The National Health and Nutrition Examination Survey (NHANES) provides an exceptional opportunity to explore these relationships on a population basis [19]. NHANES, with its nationally representative sampling and comprehensive data on health outcomes, medication use, and laboratory data, allows the prevalence of complications in GLP-1 RA users and non-users to be compared in the U.S. adult population [20].

The main objective of this study is to compare the adjusted odds of microvascular complications (defined as CKD stage ≥3, albuminuria, and diabetic retinopathy) versus macrovascular complications (defined as myocardial infarction, stroke, and coronary artery disease) among U.S. adults reporting GLP-1 receptor agonist use. In addition, the study will also establish whether the magnitude of association varies across the types of complications. By achieving this objective, the research will extend current understanding of GLP-1 RAs’ vascular effects and inform clinical decision-making and policy development for patients with diabetes and obesity.

## Materials and methods

Study design and data source

We conducted a cross-sectional analysis using data from the National Health and Nutrition Examination Survey (NHANES) 2011-2018 [21]. NHANES employs a complex, multistage probability sampling design to obtain a nationally representative sample of the noninstitutionalized U.S. population. We restricted our analysis to 2011-2018 because NHANES operations were suspended in 2019-2020 due to the COVID-19 pandemic, resulting in irregular data collection. The subsequent 2017-2020 combined cycle and 2021-2023 cycle were therefore not methodologically comparable to earlier continuous two-year cycles and were avoided to preserve data consistency and validity. Demographic, socioeconomic, clinical, and behavioral data were collected through standardized interviews, physical examinations, and laboratory assessments. All analyses incorporated survey weights, strata, and clusters to account for the complex design and ensure generalizability.

Study population and variables

The analytic sample consisted of adults aged 20 years and older with complete data on the main exposure and outcome variables. The primary exposure of interest was self-reported use of GLP-1 receptor agonists (liraglutide, semaglutide, or dulaglutide) within the past 30 days, recorded in the prescription drug questionnaire. The primary outcomes were microvascular complications (albuminuria, diabetic retinopathy) and macrovascular complications (self-reported history of myocardial infarction, stroke, or coronary artery disease). Covariates included demographic characteristics (age, gender, race/ethnicity, education level), socioeconomic status (family income-to-poverty ratio), lifestyle factors (smoking history, physical activity), and clinical measures (BMI, HDL cholesterol, and total cholesterol).

Missing data

The main exposure (GLP-1 use) and outcomes (microvascular and macrovascular complications) had no missing values. Most covariates had low levels of missingness (<10%), except the income-to-poverty ratio (10.45%) and lipid measures (10.0%). Given the complex survey design and the minimal proportion of missingness, we performed complete case analysis rather than applying multiple imputation. This approach minimized potential bias while maintaining consistency with NHANES analytic guidelines.

Statistical analysis

All analyses accounted for NHANES survey weights, strata, and primary sampling units to ensure nationally representative estimates. Descriptive statistics summarized baseline characteristics across GLP-1 receptor agonist use status. Categorical variables were compared using survey-design-based F-tests (Rao-Scott adjusted F-tests), while continuous variables were compared using survey-weighted t-tests. Logistic regression models were then applied to estimate the associations between GLP-1 use and each outcome (microvascular and macrovascular complications), adjusting for relevant covariates. To evaluate collinearity between predictors, variance inflation factors (VIFs) were calculated; all values ranged from 1.01 to 3.94, with a mean VIF of 1.81, indicating no evidence of problematic multicollinearity. All statistical analyses were conducted using Stata version 18 (StataCorp, College Station, TX).

Ethical considerations

NHANES protocols are approved annually by the National Center for Health Statistics (NCHS) Research Ethics Review Board. Written informed consent was obtained from all participants at the time of data collection. Because this study relied exclusively on de-identified, publicly available NHANES datasets, no additional institutional review board approval was required.

## Results

Table 1 below presents the baseline characteristics of U.S. adults reporting GLP-1 receptor agonist use compared with non-users, using data from NHANES 2011-2018. Overall, GLP-1 users were older, had higher body mass index (BMI), and demonstrated less favorable lipid profiles compared with non-users. Differences were also observed in the prevalence of vascular complications, while sociodemographic variables such as income, gender, smoking history, education level, physical activity, and race/ethnicity showed no statistically significant variation between groups.

**Table 1:** Baseline characteristics of U.S. adults reporting GLP-1 receptor agonist use versus non-use, NHANES 2011–2018 Data are presented as mean ± standard deviation (SD) for continuous variables and weighted counts (percentages) for categorical variables. Comparisons were made using survey-weighted t-tests for continuous variables and survey-design-based F-tests (Rao–Scott adjusted F-tests) for categorical variables. P-values <0.05 were considered statistically significant. Abbreviations: GLP-1 = glucagon-like peptide-1 receptor agonist; HDL = high-density lipoprotein; BMI = body mass index. -: Intentionally left blank.

Variable	GLP-1 non-users (%); N=230,565,440	GLP-1 users (%); N=806,155	F-test/t-test	p-value
Age in years (mean ±SD)	47.59±16.94	55.72±11.33	t= -5.34	<0.001
Body mass index (kg/m^2^), (mean ±SD)	29.29±6.97	36.39±8.27	t= -6.06	<0.001
Direct HDL-cholesterol (mg/dL), (mean ±SD)	53.87±16.53	44.80±10.99	t= 5.94	<0.001
Total cholesterol (mg/dL), (mean ±SD)	191.99±41.54	170.80±36.18	t= 3.14	<0.001
Income to poverty ratio (mean ±SD)	2.98±1.66	3.42±1.63	t= -1.62	0.111
Gender (%)	-	-	F= 0.12	0.729
Male	110,807,417(99.63%)	410,719(0.37%)	-	-
Female	119,758,022(99.67%)	395,436(0.33%)	-	-
Smoked at least 100 cigarettes in life (%)	-	-	F= 2.78	0.101
Yes	99,871,160(99.55%)	452,107(0.45%)	-	-
No	130,688,867(99.73%)	354,048(0.27%)	-	-
Any macrovascular complication (%)	-	-	F= 15.36	<0.001
Yes	18,832,409(99.11%)	169,714(0.89%)	-	-
No	211,733,030(99.70%)	636,440(0.30%)	-	-
Any microvascular complication (%)	-	-	F= 14.52	<0.001
Yes	25,558,717(99.01%)	256,610(0.99%)	-	-
No	205,006,722(99.73%)	549,545(0.27%)	-	-
Moderate-to-vigorous physical activity (%)	-	-	F= 0.32	0.709
1 -2 days	41,450,616(99.72%)	115,826(0.28%)	-	-
3-4 days	189,004,795(99.64%)	690,329(0.36%)	-	-
≥5 days	110,028(100%)	0.00(0%)	-	-
Education level - Adults 20+ (%)	-	-	F= 0.56	0.642
Less than 9th grade	11,645,373(99.74%)	30,693(0.26%)	-	-
9-11th grade	21,678,619 (99.78%)	47,062 (0.22%)	-	-
High school graduate/GED	51,869,636 (99.65%)	182,270 (0.35%)	-	-
Some college or AA degree	73,794,409 (99.68%)	233,758 (0.32%)	-	-
College graduate or above	71,577,401(99.57%)	312,371(0.43%)	-	-
Race/ethnicity (%)	-	-	F= 2.16	0.089
Mexican American	19,950,291 (99.79%)	41,305 (0.21%)	-	-
Other Hispanic	14,724,789 (99.82%)	26,145 (0.18%)	-	-
Non-Hispanic White	148,828,340 (99.61%)	580,846 (0.39%)	-	-
Non-Hispanic Black	26,287,319 (99.52%)	125,957 (0.48%)	-	-
Other race	20,774,698 (99.85%)	31,901 (0.15%)	-	-

Among continuous measures, the mean age of GLP-1 users was 55.72±11.33 years compared with 47.59±16.94 years in non-users (t = -5.34, p <0.001). Users also had a higher mean BMI (36.39±8.27 vs 29.29±6.97 kg/m², t = -6.06, p <0.001). Lipid parameters demonstrated that GLP-1 users had lower mean HDL cholesterol (44.80±10.99 mg/dL vs 53.87±16.53 mg/dL, t = 5.94, p <0.001) and lower total cholesterol (170.80±36.18 mg/dL vs 191.99±41.54 mg/dL, t = 3.14, p <0.001). The income-to-poverty ratio was slightly higher among users (3.42±1.63) compared with non-users (2.98±1.66), but this difference was not statistically significant (p =0.111).

In terms of categorical variables, gender distribution was similar between groups. Among non-users, there were 110,807,417 men (99.63%) and 119,758,022 women (99.67%), compared with 410,719 men (0.37%) and 395,436 women (0.33%) among GLP-1 users (F = 0.12, p =0.729). Smoking status did not significantly differ; 99,871,160 non-users (99.55%) and 452,107 users (0.45%) reported having smoked ≥100 cigarettes, while 130,688,867 non-users (99.73%) and 354,048 users (0.27%) reported never smoking (F = 2.78, p =0.101).

By contrast, vascular complications showed notable differences. Macrovascular complications were more prevalent among users, with 169,714 users (0.89%) reporting events compared with 18,832,409 non-users (99.11%) (F = 15.36, p <0.001). Similarly, microvascular complications were reported in 256,610 users (0.99%) versus 25,558,717 non-users (99.01%) (F = 14.52, p <0.001).

Physical activity did not differ significantly between groups. Among non-users, 41,450,616 (99.72%) engaged in 1-2 days of moderate-to-vigorous activity per week and 189,004,795 (99.64%) engaged in 3-4 days, compared with 115,826 (0.28%) and 690,329 (0.36%) among users, respectively (F = 0.32, p =0.709). None of the GLP-1 users reported ≥5 days of activity.

Education levels were comparable across groups. For example, 51,869,636 non-users (99.65%) and 182,270 users (0.35%) were high school graduates, while 71,577,401 non-users (99.57%) and 312,371 users (0.43%) were college graduates or above (F = 0.56, p =0.642).

Race and ethnicity also did not significantly differ (F = 2.16, p =0.089). Among GLP-1 users, the largest group was Non-Hispanic White with 580,846 (0.39%), followed by Non-Hispanic Black with 125,957 (0.48%), Mexican American with 41,305 (0.21%), other Hispanic with 26,145 (0.18%), and other race with 31,901 (0.15%).

Association between GLP-1 receptor agonist use and microvascular complications

Table 2 below summarizes the results of the survey-weighted logistic regression model examining the association between GLP-1 receptor agonist use and microvascular complications among U.S. adults in NHANES 2011-2018. After adjustment for demographic, socioeconomic, lifestyle, and clinical covariates, GLP-1 receptor agonist use was significantly associated with higher odds of microvascular complications.

**Table 2:** Survey-weighted logistic regression examining the association between GLP-1 receptor agonist use and microvascular complications among U.S. adults, NHANES 2011–2018 Odds ratios (ORs) and 95% confidence intervals (CIs) derived from survey-weighted logistic regression models. Microvascular complications are defined as the presence of albuminuria or diabetic retinopathy. Models adjusted for age, gender, race/ethnicity, education, income-to-poverty ratio, smoking, physical activity, BMI, HDL cholesterol, and total cholesterol. Statistical significance: * p < 0.05, ** p < 0.01, *** p < 0.001 Abbreviations: MVPA= moderate-to-vigorous physical activity -: Intentionally left blank

Predictor	Adjusted Odds Ratio (OR)	95% CI	p-value
GLP-1 receptor agonist use	-	-	-
Yes	2.29	1.05-4.97	0.037*
Age in years	1.04	1.03-1.04	<0.001***
Gender(ref: Male)	-	-	-
Female	1.11	0.95-1.31	0.192
Race/Ethnicity (ref: Mexican American)	-	-	-
Other Hispanic	0.90	0.70-1.15	0.372
Non-Hispanic White	0.83	0.67-1.02	0.077
Non-Hispanic Black	1.23	1.00-1.51	0.050
Other Race	1.24	0.98-1.55	0.067
Education (ref: < Less Than 9th Grade)	-	-	-
9-11th Grade	0.93	0.74-1.16	0.495
High School graduate/GED	0.79	0.65-0.97	0.028*
Some college or AA degree	0.76	0.61-0.94	0.012*
College graduate or above	0.69	0.54-0.88	0.004**
Income-to-poverty ratio	0.86	0.82-0.91	<0.001***
Smoked at least 100 cigarettes in life	-	-	-
Yes	0.85	0.75-0.95	0.005
MVPA (ref: 1–2 days/wk)	-	-	-
3-4 days	1.07	0.90-1.26	0.449
≥5 days	1.38	0.26-7.45	0.702
Body mass index (kg/m^2^)	1.03	1.02-1.04	<0.001***
Direct HDL-cholesterol (mg/dL)	0.99	0.99-1.00	0.017*
Total cholesterol( mg/dL)	1.00	0.99-1.00	0.944

Specifically, adults reporting GLP-1 use had 2.29 times higher odds of microvascular complications compared with non-users (OR=2.29, 95% CI: 1.05-4.97, p=0.037). Age was independently associated, with each one-year increase corresponding to higher odds of complications (OR=1.04, 95% CI: 1.03-1.04, p<0.001). Gender was not a significant predictor, as females had similar odds compared with males (OR=1.11, 95% CI: 0.95-1.31, p=0.192).

Race and ethnicity showed mixed associations. Compared with Mexican Americans, Non-Hispanic Black adults had borderline higher odds of complications (OR=1.23, 95% CI: 1.00-1.51, p=0.050), while Non-Hispanic Whites (OR=0.83, 95% CI: 0.67-1.02, p=0.077), Other Race category (OR=1.24, 95% CI: 0.98-1.55, p=0.067), and Other Hispanics (OR=0.90, 95% CI: 0.70-1.15, p=0.372) did not differ significantly.

Educational attainment demonstrated a protective effect relative to less than ninth-grade education. Adults with a high school diploma or GED had reduced odds (OR=0.79, 95% CI: 0.65-0.97, p=0.028), as did those with some college or an associate degree (OR=0.76, 95% CI: 0.61-0.94, p=0.012) and those with a college degree or higher (OR=0.69, 95% CI: 0.54-0.88, p=0.004). Income-to-poverty ratio was also inversely associated, with higher income linked to lower odds of complications (OR=0.86, 95% CI: 0.82-0.91, p<0.001).

Among lifestyle and clinical variables, smoking history was associated with reduced odds (OR=0.85, 95% CI: 0.75-0.95, p=0.005), while physical activity frequency showed no significant association. BMI was positively associated with risk (OR=1.03, 95% CI: 1.02-1.04, p<0.001). HDL cholesterol was inversely related (OR=0.99, 95% CI: 0.99-1.00, p=0.017), while total cholesterol was not significantly associated (OR=1.00, 95% CI: 0.99-1.00, p=0.944).

**Figure 1:**
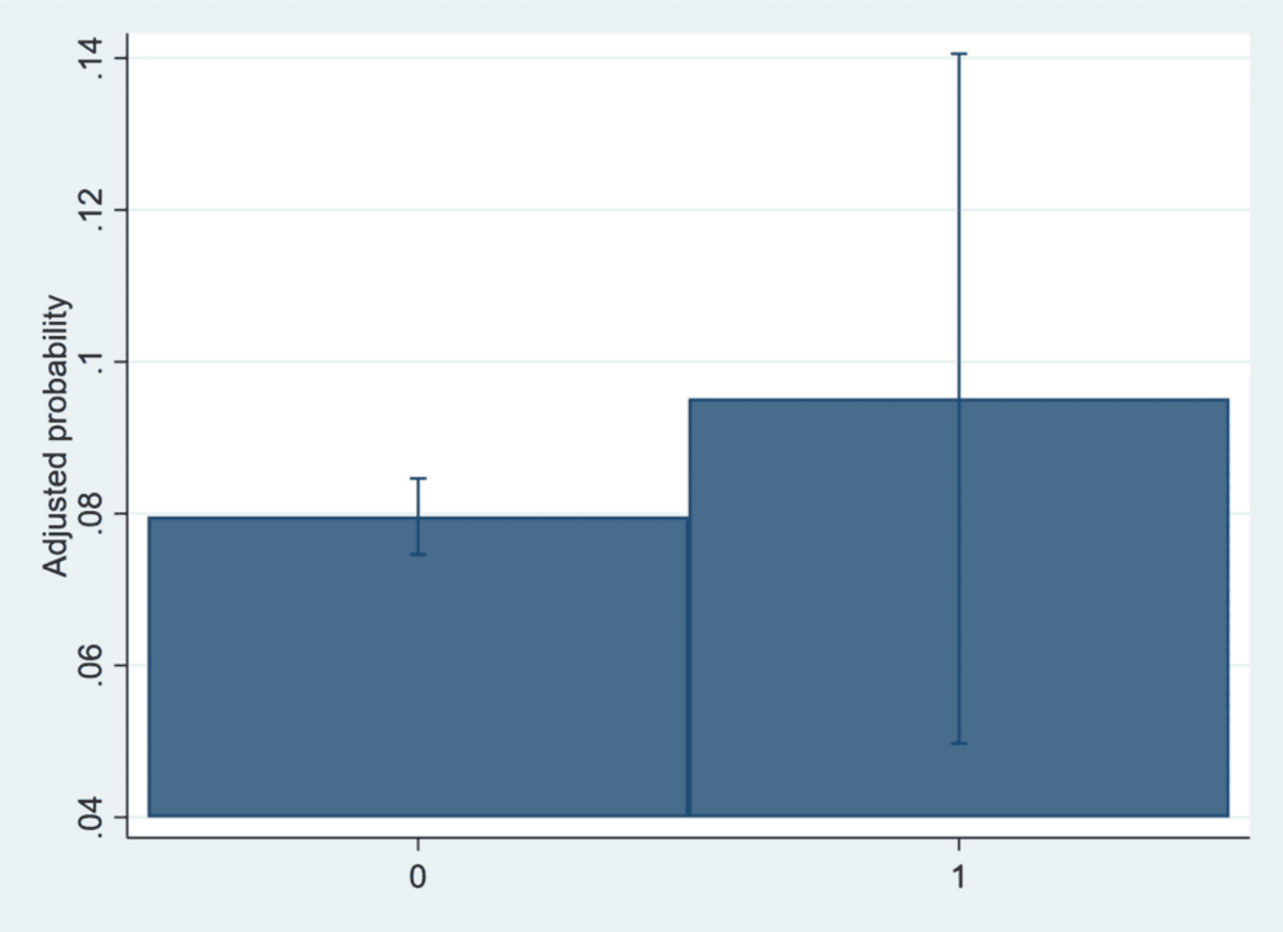
Adjusted probability of microvascular complications by GLP-1 receptor agonist use. Bars represent adjusted predicted probabilities derived from survey-weighted logistic regression models, with 95% confidence intervals. Microvascular complications were defined as the presence of albuminuria or diabetic retinopathy. Models adjusted for age, gender, race/ethnicity, education, income-to-poverty ratio, smoking status, physical activity, body mass index, HDL cholesterol, and total cholesterol. 0= GLP1-non-users, 1= GLP1 users.

To complement the regression estimates presented in Table 2, we generated adjusted predicted probabilities of microvascular complications by GLP-1 receptor agonist use. Figure 1 below displays the adjusted probabilities with 95% confidence intervals, demonstrating a higher predicted probability of microvascular complications among GLP-1 users compared to non-users.

Association between GLP-1 receptor agonist use and macrovascular complications

Table 3 below presents the results of the survey-weighted logistic regression examining the association between GLP-1 receptor agonist use and macrovascular complications among U.S. adults in NHANES 2011-2018.

**Table 3:** Survey-weighted logistic regression examining the association between GLP-1 receptor agonist use and macrovascular complications among U.S. adults, NHANES 2011–2018 Odds ratios (ORs) and 95% confidence intervals (CIs) derived from survey-weighted logistic regression models. Macrovascular complications are defined as a self-reported history of myocardial infarction, stroke, or coronary artery disease. Models adjusted for age, gender, race/ethnicity, education, income-to-poverty ratio, smoking, physical activity, BMI, HDL cholesterol, and total cholesterol. Abbreviations: MVPA = moderate-to-vigorous physical activity. Statistical significance: ** p < 0.01, *** p < 0.001 -: Intentionally left blank

Predictor	Adjusted Odds Ratio (OR)	95% CI	p-value
GLP-1 receptor agonist use	-	-	-
Yes	1.27	0.65-2.51	0.478
Age in years	1.08	1.08-1.09	<0.001 ***
Gender(ref: Male)	-	-	-
Female	0.85	0.70-1.04	0.117
Race/ethnicity (ref: Mexican American)	-	-	-
Other Hispanic	1.31	1.00-1.71	0.051
Non-Hispanic White	1.7	1.35-2.16	<0.001 ***
Non-Hispanic Black	1.93	1.48-2.53	<0.001***
Other race	1.84	1.31-2.58	0.001 ***
Education (ref: < Less Than 9th Grade)	-	-	-
9-11th grade	1.09	0.81-1.47	0.554
High school graduate/GED	0.98	0.73-1.31	0.896
Some college or AA degree	0.92	0.70-1.22	0.573
College graduate or above	0.77	0.57-1.04	0.086
Income-to-poverty ratio	0.82	0.78-0.87	<0.001 ***
Smoked at least 100 cigarettes in life	-	-	-
Yes	0.61	0.53-0.71	<0.001 ***
MVPA (ref: 1–2 days/wk)	-	-	-
3-4 days	0.99	0.81-1.22	0.939
≥5 days	0.91	0.12-7.21	0.93
Body mass index (kg/m^2^)	1.04	1.02-1.05	<0.001***
Direct HDL-cholesterol (mg/dL)	0.99	0.98-1.00	0.004 **
Total cholesterol (mg/dL)	0.99	0.99-0.99	<0.001 ***

After multivariable adjustment, GLP-1 receptor agonist use was not significantly associated with macrovascular complications (OR=1.27, 95% CI: 0.65-2.51, p=0.478).

Age showed a strong independent association, with each additional year of age increasing the odds of macrovascular complications (OR=1.08, 95% CI: 1.08-1.09, p<0.001). Gender was not significant, as females had slightly lower but statistically non-significant odds compared with males (OR=0.85, 95% CI: 0.70-1.04, p=0.117).

Race and ethnicity demonstrated significant associations relative to Mexican Americans. Non-Hispanic Whites had higher odds of complications (OR=1.70, 95% CI: 1.35-2.16, p<0.001), as did Non-Hispanic Blacks (OR=1.93, 95% CI: 1.48-2.53, p<0.001) and Other Race (OR=1.84, 95% CI: 1.31-2.58, p=0.001). Other Hispanics also had elevated odds, though borderline significant (OR=1.31, 95% CI: 1.00-1.71, p=0.051).

Educational attainment did not show consistent associations. Adults with 9-11th grade education (OR=1.09, 95% CI: 0.81-1.47, p=0.554), high school graduation/GED (OR=0.98, 95% CI: 0.73-1.31, p=0.896), and some college or associate degree (OR=0.92, 95% CI: 0.70-1.22, p=0.573) had no significant differences compared with those with less than 9th grade education. College graduates had lower odds, though this did not reach significance (OR=0.77, 95% CI: 0.57-1.04, p=0.086).

Socioeconomic status was an important factor, with a higher income-to-poverty ratio significantly reducing odds of macrovascular complications (OR=0.82, 95% CI: 0.78-0.87, p<0.001). Smoking history was also strongly associated with lower odds of complications (OR=0.61, 95% CI: 0.53-0.71, p<0.001). Physical activity showed no significant association, whether 3-4 days per week (OR=0.99, 95% CI: 0.81-1.22, p=0.939) or ≥5 days (OR=0.91, 95% CI: 0.12-7.21, p=0.930), relative to 1-2 days per week.

Clinical factors revealed that BMI was positively associated with macrovascular complications (OR=1.04, 95% CI: 1.02-1.05, p<0.001). HDL cholesterol was inversely associated (OR=0.99, 95% CI: 0.98-1.00, p=0.004), while total cholesterol showed a statistically significant but inverse relationship, with higher levels associated with lower odds of complications (OR=0.99, 95% CI: 0.99-0.99, p<0.001).

## Discussion

In this nationally representative analysis of U.S. adults using NHANES 2011-2018 data, we found that GLP-1 RA use was not significantly associated with lower odds of macrovascular complications, whereas the use of GLP-1 RAs was linked to higher odds of microvascular complications following adjustment for confounders. These findings contrast with prior cardiovascular outcome trials (CVOTs) and meta-analyses that demonstrated robust benefits of GLP-1 RAs on major adverse cardiovascular events but also suggested heterogeneity depending on the population and outcome examined [14, 15]. The reported association with increased odds of microvascular complications potentially indicates that individuals prescribed GLP-1 RAs had increasingly higher underlying risk profiles for the microvascular complications (a palpable instance of confounding by indication), and this has been supported by the baseline characteristics noted in Table 1 indicating that GLP-1 users presented higher BMIs in addition to having significantly higher unadjusted prevalence with regard to the micro- and macrovascular complications. 

Our results reinforce the notion that GLP-1 RAs may provide clinically meaningful benefits beyond glucose lowering, consistent with evidence of pleiotropic effects such as weight reduction, lipid modulation, and anti-inflammatory activity [12, 13, 15]. Importantly, the potential preferential protection against microvascular outcomes may have practical relevance, as these complications often manifest earlier in the disease course and substantially contribute to morbidity and health care burden [9, 10].

Furthermore, obesity is known as one of the risk factors for insulin resistance, which ultimately leads to high blood glucose levels (hyperglycemia) [1]. Hyperglycemia triggers non-enzymatic glycation of collagen throughout the body, mostly in blood vessels [6]. Non-enzymatic glycation of proteins can occur both intracellularly and extracellularly, leading to the formation of advanced glycation end products (AGEs) [6]. When circulating proteins such as albumin become glycated in the plasma, they stimulate various receptors on endothelial cells of blood vessels (microvascular and macrovascular), leading to endothelial dysfunction [6]. Endothelial dysfunction results from the accumulation of AGEs over time, which induces inflammation and oxidative stress within endothelial cells through interaction with plasma membrane-localized receptors for AGEs [6]. This process triggers intracellular signaling that promotes gene expression, the release of free radicals, and pro-inflammatory molecules [6, 7]. Also, the cumulative effects of the AGE-mediated endothelial injury are a major contributor to various long-term clinical implications related to diabetes, including atherosclerosis, an increase in vascular stiffness, and specific macro- and microvascular events assessed in our study. Obesity’s role in driving the pathogenic cascade is of immense clinical significance in patient populations with conditions like HIV and congestive heart failure. In such persons, obesity frequently coexists and worsens disease progression via metabolic dysregulation and severe inflammation, which may further accelerate AGE formation and vascular damage [8]. Therefore, the management of obesity in such intricate multimorbid patients is a vital therapeutic target to enhance cardiometabolic outcomes. Many studies have emphasized the pharmacotherapeutic effects of various medications in minimizing vascular damage. GLP-1 RAs have not only demonstrated metabolic effects, such as tight glycemic control, but have also recently been recognized for their anti-inflammatory effects by reducing the formation of advanced glycation end products [15]. The anti-inflammatory and weight-reducing properties of GLP-1 RAs might be particularly advantageous for individuals with obesity complicating conditions such as HIV and congestive heart failure, providing a complex approach to reducing their elevated risk of cardiovascular disease [1, 4-8].

The analysis confirmed numerous expected relationships in addition to disclosing an increasingly complex relationship with lipid parameters. Thus, the positive association existing between macrovascular complications and BMI is consistent with extensive literature that connects obesity to adverse cardiovascular outcomes, potentially mediated by inflammation, endothelial dysfunction, and insulin resistance. Furthermore, despite being statistically significant, the inverse correlation between HDL cholesterol and complications had an increasingly modest effect size (OR = 0.99 per unit increase). This proposes that, despite HDL’s protective role being present, its independent effect on the study cohort, which already had established dyslipidemia, might be limited. Additionally, a paradoxical and significant inverse relationship between the macrovascular complications and total cholesterol levels was observed. Such a counterintuitive finding is possibly not causal but somewhat an instance of confounding through reverse epidemiology or indication. Patients with higher cholesterol are increasingly prone to be prescribed and constantly use potent statin therapy, which has been acknowledged to drastically reduce LDL and total cholesterol, even as it simultaneously lowers cardiovascular risk. Consequently, the measured lower total cholesterol in several patients with complications might be a result of intensive lipid-reduction treatment as opposed to the cause of their reduced risk.

Various studies have shown that dietary risk accounts for 26% of deaths and 14% of disability-adjusted life years in obese people [3,4, 7]. Improvements in diet represent a huge potential for disease reduction either directly or indirectly through improvements in intermediate risk factors such as blood pressure, fasting glucose, and weight gain [4]. Obesity produces a variety of structural and functional adaptations to the cardiovascular system, such as lowering the cardiac output, increased peripheral resistance, and poorer left ventricular systolic function [4]. Obesity is also known to influence coronary risk indirectly through its effect on related comorbidities such as dyslipidaemia, hypertension, glucose intolerance, endothelial dysfunction, and inflammation [4]. In these regards, GLP-1 agonist has demonstrated significant weight reduction benefits [12], a double-blinded trial conducted on participants with BMI of 30 or more without T2DM or BMI of 27 or more with treated or untreated dyslipidemia or hypertension showed that 63.2% of patients in the GLP agonist group lost at least 5% of their body weight compared to the 27.1% in the placebo group [12]. Also, 33.1% and 10.6% respectively lost more than 10% of their body weight [12]. One of the cardiovascular benefits of GLP-1 agonists includes cardioprotection in DM patients [12]. Similarly, in non-diabetic patients, they have also been shown to reduce cardiovascular events and mortality [12]. Semaglutide has been shown to reduce the risk of death from non-fatal myocardial infarction or stroke by 20% due to its weight reduction properties [12]. GLP-1 agonists have also been shown to reduce fatty acid synthesis in the liver, thereby further decreasing LDL and total cholesterol levels while increasing HDL [12]. Some of the microvascular complications, such as diabetic nephropathy, are reduced by GLP-1 agonists through reduced renal tubular injury and decreased tubulointerstitial damage [12].

Strengths and limitations of the study

A key strength of this study is the use of NHANES data, which is nationally representative and allows for generalizable estimates across diverse sociodemographic subgroups. The survey-weighted design and multivariable adjustment for demographic, socioeconomic, and clinical covariates reduce the risk of bias, enhancing the robustness of the findings. Additionally, the simultaneous evaluation of both microvascular and macrovascular endpoints provides a more refined perspective on the differential vascular impact of GLP-1 RAs. Nevertheless, several limitations merit consideration. First, GLP-1 RA use was relatively uncommon in the study period (2011-2018), reflecting their limited availability and adoption at the time [19, 20]. This low prevalence likely reduced statistical power, particularly for subgroup analyses, and may explain the lack of significant findings for macrovascular endpoints. Second, the cross-sectional design of NHANES precludes causal inference and does not capture temporal relationships between drug exposure and outcomes. Third, outcome ascertainment relied partly on self-reported diagnoses, which may introduce misclassification bias. Finally, although models were adjusted for multiple covariates, the possibility of residual confounding cannot be excluded. Future studies with more contemporary NHANES cycles and larger samples of GLP-1 RA users are warranted to validate these findings, particularly given the exponential growth in GLP-1 RA prescribing after 2018. Longitudinal and registry-based studies should also clarify temporal patterns and explore whether protective associations differ by specific GLP-1 agents, dosing, or treatment duration.

## Conclusions

In this nationally representative analysis, GLP-1 receptor agonist use was more strongly associated with protection against microvascular than macrovascular complications. This study offers a unique, real-world evidence drawn from the general United States population that further supports the increasing evidence from various trials, indicating that GLP-1 RAs might offer benefits beyond glycemic control, especially for renal outcomes. However, given the observational nature and the limited use of GLP-1 RAs during the study period, further contemporary and longitudinal research is needed to confirm these associations and better define their role in preventing both microvascular and macrovascular complications.
